# Orthopaedic Injuries in 272 Dressage Horses: A Retrospective Study

**DOI:** 10.3390/ani15202972

**Published:** 2025-10-14

**Authors:** Ana Boado, Danica Pollard, Francisco Javier Lopez-Sanroman, Sue Dyson

**Affiliations:** 1 Independent Researcher, Avenida Salmoral 4, Manzanares el Real, 28492 Madrid, Spain; 2 Medicine and Surgery Department, Universidad Complutense de Madrid, Av. Complutense, Moncloa-Aravaca, 28040 Madrid, Spain; lsroman@ucm.es; 3 Independent Researcher, Rodham Road, Christchurch, Wisbech, Cambridgeshire PE14 9NU, UK; drdee.pollard@gmail.com; 4 Independent Researcher, Church Road, Market Weston, Diss, Suffolk IP22 2NX, UK; sue.dyson@aol.com

**Keywords:** lameness, poor performance, Warmbloods, Purebred Spanish Horses (PRE), Lusitano horses, clinical examination, diagnostic anaesthesia, radiography, ultrasonography

## Abstract

**Simple Summary:**

Information about orthopaedic injuries in dressage horses is limited. Two hundred and seventy-two dressage horses, mostly Warmbloods (58.5%) and Iberian horses (38.6%) underwent comprehensive orthopaedic examinations, including ridden exercise. The most frequent lameness grade (0–5 scale) was 2 (range 0–4). There was a high prevalence of multilimbed lameness (44.9%) and multifocal pain (46.6%) causing lameness, but there was no difference in outcome compared with single-source pain unilateral lameness. Forelimb lameness (72.1%) occurred more frequently than hindlimb lameness (27.9%). Foot (39.7%), fetlock (29.4%) and metacarpal/metatarsal (32.0%) pain were the most common causes of lameness. Forelimb proximal suspensory desmitis (20.6%), hindlimb proximal suspensory desmopathy (1.5%) and suspensory branch injury (11.8%) were the most common soft tissue injuries. Spinal pain was identified in 21.7% of horses, mainly affecting thoracolumbar (9.6%) and lumbosacroiliac (9.9%) areas or both (2.9%). Of 238 horses for which long-term (one to five years after injury) follow-up was available, 42.4% resumed full work at the same level as pre-injury or higher, after targeted treatment and management. The level at which a horse had competed pre-injury, age, breed, sex and lameness grade had no effect on follow-up outcome. Accurate diagnosis and appropriate treatment may enable dressage horses to return to full athletic function.

**Abstract:**

There is limited information regarding orthopaedic injuries in dressage horses. This study assessed the prevalence of injuries in a mixed referral and first opinion population of 272 horses training and competing in dressage, 238 of which were followed up one to five years after injury. Warmblood (55.8%) and Iberian (38.6%) breeds predominated. The median age was 8 years (interquartile range [IQR] 5,11; range 1,21). Horses were examined due to lameness (85.3%) or poor performance (14.7%). The median lameness grade was 2/5 (IQR 2,4; range 0,4). Forelimb lameness was more prevalent than hindlimb lameness (*p* < 0.001). Unilateral lameness (55.2%) was more common than multilimbed lameness (44.9%). Hypermetria (14.3%), hypermetria and weakness (2.2%) or weakness alone (1.1%) did not affect follow-up outcome compared with horses without neurological gait dysfunction. Soft tissue injuries were more common in Iberian horses than in Warmbloods (*p* = 0.006), but considering all injuries there was no difference in outcome between breeds. Injury type (soft tissue, osseous, osteoarthritis, developmental disease) did not influence follow-up outcome. Following targeted treatment and tailored rehabilitation programs, 42% of 238 horses returned to the same level of work as pre-injury or higher, 45% returned to work at a lower level and 13% were retired due to orthopaedic injury. In-depth clinical assessment and diagnostic anaesthesia are important for the identification of all problems and development of the most appropriate treatment and management protocols.

## 1. Introduction

Orthopaedic injuries are the main cause of poor performance in horses [1]. The prevalence of injuries may be related to work discipline and the level at which a horse is training and competing [2]. The risk factors for orthopaedic injuries related to dressage have been investigated in three studies, two of which relied on owners’ responses to a questionnaire [3,4] and the third was based on a referral population of dressage horses [2] rather than those seen in first opinion practice.

Although in low levels of dressage the biomechanical demands on horses are theoretically similar to those of other sports horses, at high levels (Prix St Georges and above) dressage horses are subjected to unusual biomechanical loads while performing exercises unique to this discipline [5,6,7]. For instance, collected trot increases stride duration compared with working trot [5], with greater elevation of the withers and a decrease in pelvic height [6] compared with working trot. This requires greater flexion and more propulsion from the hindlimbs; the forelimbs must exert increased push off to elevate the forehand into the suspension phase [6]. Moreover, given the commercial pressures of the industry and the current movement patterns favored by judges, breeders are encouraged to produce horses with inherent flamboyant movement patterns (for example, advanced diagonal placement in trot) [7]. Three- and four-year-old horses are often produced for young horse classes, sales, and stallion grading, when they have insufficient musculoskeletal strength and coordination to support the movements that they are asked to perform, with an inherent injury risk, particularly repetitive strain type injuries [8,9].

Exercises that require collection such as pirouettes, flying changes, passage and piaffe require rapid flexion and extension of the thoracolumbosacral region [10]. Lumbosacral flexion is maximal in some exercises, such as piaffe, increasing forces over the sacroiliac and lumbosacral joints [7,10,11,12,13].

Lateral movements such as shoulder-in, travers, renvers and half-pass involve lateral flexion and rotation of the vertebral column, and weight bearing is asymmetric during these exercises. Therefore, horses are subjected to different loads and stresses compared with natural gaits, when horses are moving straight on two tracks (the hindlimbs following the tracks of the forelimbs). Extended trot increases fetlock extension compared with collected trot and thus increases load on the suspensory apparatus [10,14]. Although training for these movements should be slowly progressive, with cross-training to avoid repetitive overload, commercial pressures mean that often this is not the case. The Fédération Equestre International governs international dressage competitions. International dressage competitions for young horses, 5 and 6 years of age, are the basis for higher level competition, such as Prix St. Georges, Intermediate I and II and ultimately Grand Prix [15]. Horses are not allowed to compete at the highest level (Grand Prix) until 8 years of age, which was raised to 9 years in 2025. Each National Federation has different categories for horses competing at lower levels nationally.

Warmblood breeds predominate in dressage throughout the world, although Iberian horses (Purebred Spanish Horses [PRE] and Lusitanos) have been increasing in popularity. The innate movement patterns of these two groups of horses are different [16]. This, together with genetic predisposition to injury, may result in differences in the prevalence of specific injuries [17,18,19].

The objectives of the study were as follows. 1. Determine the prevalence of different orthopaedic injuries in dressage horses. 2. Determine the frequency of injuries occurring at a single site in one limb, or in more than one anatomical location in a single limb, or concurrent injuries occurring in more than one limb. 3. Describe the prevalence of injuries in Iberian horses compared with Warmblood horses.

It was hypothesized that, 1. the presence of more than one injury site would not affect prognosis; 2. lesion prevalence would not be different in Iberian horses compared with Warmbloods; and 3. horses that had paddock turn out prior to injury would be more likely to return to full function at the same level or higher compared with horses that did not have paddock access.

## 2. Material and Methods

### 2.1. Data Collection

The clinical records of orthopaedic examinations of 272 horses that were in training for or competing in dressage were reviewed. This did not include those horses undergoing routine examinations which have been previously reported [20]. All horses were examined by one experienced clinician, a Diplomate of the European and American Colleges of Veterinary Sports Medicine and Rehabilitation. Horses were presented for investigation of lameness or poor performance between 2008 and 2023 and included both first opinion cases and horses referred for specialist examination. Most of the examinations were performed in Spain, except when horses were competing abroad for extended periods.

A comprehensive history was obtained at the examination including age, sex and breed, reason for examination, the level at which the horse was working, specific difficulties the rider had experienced, and any prior history of orthopaedic injuries or observations made at a previous pre-purchase examination. Other diseases and known surgical interventions were also recorded. The latter information influenced aspects of the clinical and imaging examinations. The level at which a horse was working/competing was classified as 1. young horses (horses up to 7 years of age) and amateurs (riders and horses working below Prix St Georges), 2. Prix St Georges, 3. Intermediate I and II and International classes for riders under 25 years of age (U25), and 4. Grand Prix, reflecting the division of horses in Spain.

The ridden exercises in which the owner, trainer or rider had most difficulties were categorised. If a horse had problems in more than one category, for the purposes of this study it was included in the most clinically relevant category.

Collection: difficulties with collection (including piaffe and passage, when required) and/or transitions, but comfortable in working, medium and extended paces.

Extended trot: if a horse refused to extend the stride or signs of pain became more evident during extended trot compared with working or medium trot.

Lateral exercises: lameness or behavioral signs of pain [21] deteriorated while performing lateral work (for example, shoulder-in, renvers, travers, half-pass).

Canter: a horse was reluctant to canter or showed behavioral signs of pain, lack of engagement, close temporal and spatial placement of the hindlimbs, incorrect lead, became disunited or had difficulties performing flying changes (single or sequence changes) or canter pirouettes.

### 2.2. Orthopaedic Examinations

All horses were initially examined in a stable, to evaluate demeanour, posture, conformation, body condition, muscle symmetry, hoof capsule shape and size and behaviour. Systematic palpation of all four limbs, the cervicothoracic, thoracolumbosacral and pelvic regions was performed [22]. Range of motion and reaction to passive distal and proximal limb flexion of the forelimbs and hindlimbs were evaluated. Epaxial muscle tone, reaction to palpation and range of motion of the thoracolumbosacral region were assessed.

Dynamic examination was performed initially on a hard surface in straight lines at the walk and trot, observing from behind, in front and from the side. Lameness was graded on a scale of 1–5 (0 = non-lame, 1 = subtle, 2 = mild, 3 = moderate, 4 = severe, 5 = non-weight bearing), under each circumstance in which a horse was examined (in hand, on the lunge on each of soft and firm surfaces, and ridden). Any variations in limb flight or limb placement were recorded. Flexion tests (distal limb flexion in the forelimbs and distal and proximal limb flexion, independently, in the hindlimbs, each for 45 s) were performed in most of the examinations, unless contraindicated by the prior history, a horse’s behaviour or at an owner’s request. Flexions tests were performed in the least lame limb first. Responses to flexion were recorded as negative, mild, moderate or severe.

Horses were lunged on a circle of approximately 20 m diameter on a soft surface and then on a hard surface, when it was safe to do so and with the owner’s agreement. Ridden examination was performed whenever possible (unless the horse was too lame, or a soft tissue injury was suspected based on the initial examinations, or a rider or suitable facilities were unavailable), with each horse evaluated working through its full repertoire of movements at the level at which it was working. The rider was asked to work the horse as they would normally do, to include rising and sitting trot, and to perform the movements in which the rider or trainer had previously noted compromised performance. If lameness was evident only when performing a specific movement this was recorded.

If the lameness grade was severe when a horse was examined in hand or on the lunge, or deteriorated as the horse trotted, diagnostic imaging was performed, directed by the initial clinical examination. Ultimately all horses underwent diagnostic imaging.

In addition to lameness assessment the gait was also classified as neurologically normal, hypermetric (increased range of motion), weak or hypermetric and weak [23,24]. Hindlimb weakness was defined as an inability to support weight on one hindlimb, limited resistance to tail pull and the inability to counteract inertia on a turn.

### 2.3. Diagnostic Anaesthesia

Perineural and intra-articular anaesthesia were performed to determine the source(s) of pain [25], unless it was clinically contraindicated to do so. In horses with lameness in more than one limb, nerve blocks were first performed in the lamest limb, followed by other lame limbs as clinically indicated. Lameness was regraded after each local anaesthetic technique.

### 2.4. Diagnostic Imaging

Diagnostic imaging (radiography and ultrasonography) was directed by the results of the initial clinical assessment and the results of nerve blocks, where applicable. Standard radiographic imaging sequences were acquired (Supplementary Table S1) [26], with additional views as required, using portable radiographic equipment (Sound-Eklin, Grand Rapids, MI, USA followed by Varex DR, XR PAD 2HR 100 uM pixel, Varex Imaging, Pioneer Road, Salt Lake City, UT, USA from 2019 onwards). Ultrasonography was performed using a Z One, (Zonare Medical Systems, Mountain View, CA, USA) with a linear transducer, 4–12 MHz, and transverse and longitudinal images were acquired. At sequential examinations any previously identified soft tissue lesions were monitored ultrasonographically. When clinically indicated, skeletal scintigraphy, magnetic resonance imaging or computed tomography were performed at several referral centers.

### 2.5. Injury Classification

#### 2.5.1. Forelimb and Hindlimb Pain

The source(s) of forelimb and hindlimb pain was divided into anatomical areas: the foot, the fetlock region (including the metacarpophalangeal or metatarsophalangeal joints, the digital flexor tendon sheath, the superficial [SDFT] and deep [DDFT] digital flexor tendons, the suspensory ligament branches and the proximal half of the straight and oblique sesamoidean ligaments), the metacarpal and metatarsal regions (including body and origin of the suspensory ligament, SDFT, DDFT, accessory ligament of the DDFT [AL-DDFT] and the metacarpal or metatarsal bones), the carpus or tarsus, and proximal limb lameness (including humeroradial, scapulohumeral, stifle [femorotibial and femoropatellar] and coxofemoral regions).

If several anatomical regions within a limb were affected concurrently, the lameness was designated as multifocal. If there was more than one lame limb, the lameness was described as multilimbed.

#### 2.5.2. Injury Type

Injuries were classified as follows:

- Osteoarthritis based on definitive radiological abnormalities [21] or the presence of osteophytes, periarticular new bone formation or modelling detected using ultrasonography (sacroiliac and lumbosacral joints);
- Soft tissue based on ultrasonographic abnormalities of ligaments, tendons, or muscles;
- Bone when osseous tissue was considered the primary source of pain;
- Developmental disease, including osteochondrosis and compression of incompletely ossified bones, and periarticular modelling (for example, osteophytes or entheseophytes) in horses less than four years of age.

#### 2.5.3. Foot Pain

Within the foot there were three categories of injury, firstly dorsal including pain localized to the distal interphalangeal joint and/or collateral ligaments and injuries of the ungular cartilages. Secondly, palmar foot pain which was defined as abnormalities of one or more of the following structures: the navicular bone, the collateral sesamoidean ligaments, the distal sesamoidean impar ligament and its insertion on the distal phalanx, the navicular bursa and the deep digital flexor tendon. Thirdly, a combination of injuries, when several areas were affected, were recorded.

Dysfunction of the suspensory apparatus of the distal phalanx which was characterized by rotation of the distal phalanx (i.e., lack of parallelism between the dorsal hoof wall and dorsal aspect of the distal phalanx) was recorded to assess if there was any impact on outcome.

#### 2.5.4. Cervicothoracolumbosacral Region

The axial skeleton was divided into cranial cervical (occiput—fourth cervical vertebra [C4], but excluding entheseous new bone at the insertions of the semispinalis muscle and nuchal ligament), caudal cervical (C5—first thoracic vertebra [T1]; this included both caudal neck pain or forelimb lameness attributed to cervical radiculopathy), thoracolumbar (T1—sixth lumbar vertebra [L6]) and lumbosacroiliac regions.

Spinal pain was considered primary when the main reason for poor performance was localized to this area by either clinical signs, regional anaesthesia, or a response to local treatments in those horses in which diagnostic anaesthesia was not performed.

### 2.6. Treatment and Outcome

Treatments are summarized in Table 1. Rehabilitation programs were adapted to individuals and initial lesion severity and progress after treatment. Intra-articular treatments, or regional spinal injections (plus radial pressure wave therapy for severe thoracolumbar pain) were normally followed by 48 h of hand-walking exercise and restriction of the most painful exercises for each horse for the following 15 days. Soft tissue injuries were monitored at six-weeks’ intervals to assess response to treatments.

In all horses, foot imbalance was corrected as far as possible and recommendations for shoeing were made in association with a farrier. Horses were always reevaluated after trimming and shoeing to be sure that lameness was stable or improved. If lameness deteriorated, then the shoes were changed accordingly.

Follow-up information was based upon competition records for 1–5 years after diagnosis and by direct telephone contact with owners. Outcome was classified into three categories: returned to full function at the same level as pre-injury or higher, returned to training at a lower level than pre-injury, and retirement from competition or training as the result of the injury.

### 2.7. Data Analysis

Data were stored in a Microsoft Excel spreadsheet (Office 365; Microsoft Corporation, Redmont, WA, USA) and imported into R Statistical Software (v4.4.2; R Foundation for Statistical Computing, Vienna, Austria. https://www.R-project.org) for all descriptive and inferential statistical analyses. Horse age (years) was not normally distributed (Shapiro–Wilk test *p* < 0.05) and lameness grade was an ordinal variable thus both were described using medians with corresponding interquartile range (IQR) and range. Lameness grade was also categorized as a binary variable (0–2, ≥3), to increase statistical power. The rest of the variables such as breed (Warmblood, Iberian, other) and sex (female, gelding, stallion), dressage level (young or amateur, Prix St Georges, Intermediate I and II and U25, Grand Prix), access to a paddock (yes or no), reason for clinical examination (lameness or poor performance), location of lameness (forelimb, hindlimb, bilateral, spine), exercise type (canter, collection, extended trot, lateral movement), presence of neurological gait abnormality (normal, hypermetric, weak, hypermetric and weak), lameness category (non-lame, subtle, mild, moderate, severe), presence of multifocal pain causing lameness, presence of multilimbed lameness, injury region, injury type (osteoarthritis, soft tissue, bone, developmental), if advanced imaging was performed and follow-up outcome (returned to same level of performance or higher, decreased level of performance, retired; also recategorized to a binary variable as good and poor/bad), were described as proportions and expressed as percentages.

A two-sample test for equality of proportions (without a continuity correction) based on the Z-test was used to compare the proportion of forelimb and hindlimb lameness, left forelimb and right forelimb lameness and left hindlimb and right hindlimb lameness.

The relationship between follow-up outcome and signalment, dressage level and lameness-related variables was assessed using Pearson’s Chi-squared (*Χ*^2^) test (with Yate’s continuity correction) or the Fisher’s exact test (when any one observed frequency in the contingency table ≤ 5). Cramér’s V and corresponding 95% confidence intervals (CIs) was calculated to estimate the effect size (ES) measure for the *Χ*^2^ test. The strength of association for a *Χ*^2^ test with degrees of freedom (df) = 1 was interpreted as weak when ES < 0.30, moderate if 0.30 < ES < 0.50 and strong when ES ≥ 0.50 as suggested by Cohen [27]. The strength of association for a *Χ*^2^ test with df = 2 was interpreted as weak when ES < 0.21, moderate if 0.21 < ES < 0.35 and strong when ES ≥ 0.35. Pairwise comparisons were carried out post hoc on categorical variables with more than two levels and where the *Χ*^2^ or Fisher’s exact test *p* < 0.05, to identify exactly which levels were different (including Bonferroni adjustment for multiple comparisons).

The relationship between breed and injury region and injury type, and spinal pain and exercise type, dressage level and presence of neurological gait abnormality was assessed using *Χ*^2^ test or the Fisher’s exact test as detailed above. For these breed comparisons, only Iberian and Warmblood horses were retained for comparison due to the low number of other breed categories.

The relationship between follow-up outcome and lameness grade and horse age, and lameness grade and dressage level and reason for clinical examination, was assessed using the Kruskal–Wallis rank sum test. A post hoc Dunn test with Bonferroni adjustment was used when variables had more than two categories to identify pairwise differences between each category. The eta-squared measure was calculated to estimate the ES based on the H-statistic [27] and ES < 0.06 was considered weak, 0.06 < ES < 0.14 was considered moderate and ≥0.14 was considered strong.

A significance threshold of *p* ≤ 0.05 was used without adjustment for multiple comparisons [28].

## 3. Results

### 3.1. Horse Signalment and Work Level

**Figure 1:**
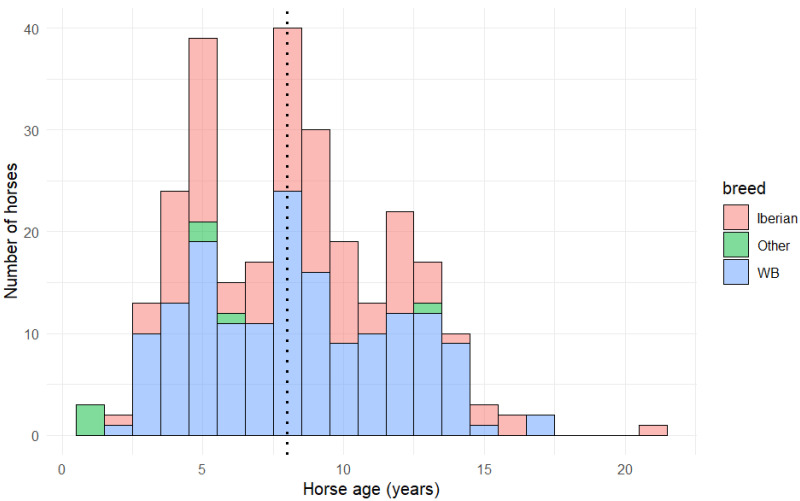
Distribution of ages by breed in 272 dressage horses examined for lameness, pain or reduced performance. The black dotted line represents the overall median age (8 years); there was a difference in median age between Warmbloods (WB) and other breeds (Dunn test *p* = 0.05 [with Bonferroni adjustment] but not between WB and Iberian breeds (Dunn test *p* = 1.00) or between Iberian and other breeds (Dunn test *p* = 0.07).

Breeds comprised 160 (58.8%) Warmbloods, 105 (38.6%) Iberian (PRE, n = 88 and Lusitano, n = 17) and 7 cross breed (2.6%). There were 130 (47.8%) geldings, 99 (36.4%) stallions and 43 (15.8%) mares. The median age at the time of investigation was 8 years (IQR 5,11; range 1,21 years). However, there were additional peaks at five and nine years of age (Figure 1).

There were 178 (65.4%) amateur and young horses, 68 (25%) training or competing at Prix St Georges, 16 at Intermediate I and/or II and U25 (5.9%) and 10 (3.7%) horses at Grand Prix. None of the amateur or U25 horses were ‘schoolmasters’ that had been downgraded from higher levels of competition.

### 3.2. Orthopaedic Examination

#### 3.2.1. Reason for Initial Examination and Lameness Grades

The reason for the initial examination was because of swelling, and/or lameness in 232 (85.3%) horses or decreased performance in 40 (14.7%) horses. Lameness grade was 0 in 35 (12.9%) horses, grade 1/5 in 18 (6.6%), 2/5 in 153 (56.3%), 3/5 in 63 (23.2%) and 4/5 in 3 (1.1%). Median lameness grade was 2/5 (IQR 2,4). Horses examined for poor performance had a lower worst lameness grade (median 1; IQR 0,1.25) compared with horses examined for lameness (median 2; IQR 2, 3; range 0, 4) (*p* < 0.001, ES = 0.20 [large effect]). Thirty-four (85%) horses examined for poor performance were lame during the orthopaedic examination. Horses with lameness grade ≥ 3 had a worse outcome than horses with lower grades, although this was not statistically significant (*Χ*^2^ = 5.0, *p* = 0.078; ES = 0.11, 95% CI 0.0, 0.3 [weak effect]).

#### 3.2.2. Distribution of Lame Limbs

Forelimb lameness (72.1%) was more prevalent than hindlimb lameness (27.9%) (*p* < 0.001). Left forelimb lameness was present in 158 (58.1%) of the horses, right forelimb lameness in 141 (51.8%), left hindlimb lameness in 53 (19.5%) and right hindlimb lameness in 54 (19.9%). Bilateral forelimb lameness was present in 103 (37.9%) horses and bilateral hindlimb lameness in 31 (11.4%) horses. The total exceeds 272 because of the presence of lameness in more than one limb in 122 (44.9%) horses, whereas single limb lameness was present in 150 (55.2%) horses. Several areas of pain in the same limb were detected in 127 horses (46.7%). The most common combinations were pain localized to the fetlock region and metacarpal pain in 41 horses (15.1% of the total population), and foot and metacarpal pain in 27 horses (9.9%). Neither multifocal sources of pain, nor multilimbed lameness had a significant impact on follow-up outcome.

#### 3.2.3. Neurological Dysfunction

Hypermetric gaits were detected in 39 (14.3%) horses, and six (2.2%) showed hypermetria and weakness. Two horses (1.1%) only exhibited weakness.

#### 3.2.4. Ridden Exercise

Ridden examination was performed in 64 (23.6%) animals, and the most common problems were detected during collection in 43/64 horses (67.2%), followed by canter exercises in 9/64 horses (14.1%), lateral exercises in 7/64 (10.9%) and extended trot in 5/64 horses (7.8%).

### 3.3. Anatomical Site of Injury

#### 3.3.1. Foot Pain

Foot pain was present in 108 (39.7%) of horses; pain was localised to the fetlock region in 80 (29.4%) horses, the metacarpal/metatarsal region in 87 (32.0%), the carpal or tarsal regions in 26 (9.5%) and the proximal aspect of a limb in 17 (6.2%). Spinal problems were identified in 59 (21.7%) horses.

Within the foot region 65/108 (60.2%) had injuries in the dorsal region, 20/108 (18.5%) in the palmar region and there was a combination of injuries in 23/108 horses (21.3%).

Specific diagnoses are summarized in Table 2.

#### 3.3.2. Fetlock, Metacarpal and Metatarsal Region Injuries

There was an association between metacarpal/metatarsal region injuries and fetlock region pain (*Χ*^2^ = 18.1, *p* < 0.001; ES = 0.26, 95% CI 0.13, 0.38 [weak effect]). Horses with fetlock region pain were more likely to have MC/MT injuries (51.2%) compared with horses with no fetlock region pain (24.0%). Metacarpal/metatarsal injuries were more prevalent in Iberian than Warmblood horses (37.1% versus 28.7%). The types of soft tissue injury in the metacarpal and metatarsal and fetlock regions are summarized in Table 3.

#### 3.3.3. Spinal Injuries

Within the spinal regions, cranial cervical injury combined with caudal cervical pain were detected in one out of 59 horses, caudal cervical pain in 15/59 horses (25.4%), thoracolumbar region pain in 26/59 horses (44.1%), lumbosacroiliac region pain in 27/59 horses (45.8%), and both thoracolumbar and lumbosacroiliac regions in 8/59 horses (13.6%). In total there were nine horses with multifocal spinal pain. Of the horses with thoracolumbar and lumbosacroiliac region pain 6/59 horses (10.9%) had difficulties during canter and 22/55 (40.0%) during collected exercises. Five of six horses with canter difficulties had lumbosacroiliac region pain, four of which had concurrent forelimb lameness, and one had concurrent hindlimb lameness. The prevalence of all regions of spinal pain was 40.0% at Grand Prix level, 21.3% at young and amateur level, 20.6% at Prix St Georges and 18.8% at Intermediate and U25.

#### 3.3.4. Neurological Dysfunction

There was an association between spinal pain and hypermetria and/or weakness (*p* = 0.003). Horses with neither hypermetria nor weakness had a lower proportion of spinal pain compared with horses with hypermetria and/or weakness (*p* = 0.007). There was an association between abnormalities related to the caudal cervical region and hypermetria and/or weakness (*p* < 0.001). Horses with neither hypermetria nor weakness had a significantly lower proportion of abnormalities related to the caudal cervical area (post hoc *p* = 0.016), while horses with hypermetria and weakness (*p* < 0.001) or a weak gait alone (*p* = 0.004) had a higher proportion of abnormalities related to the caudal cervical region.

### 3.4. Osteoarthritis, Soft Tissue Injury, Bone Injury and Developmental Disease

Osteoarthritis was present in 144/272 (52.9%) horses (a summary of the prevalence per joint is described in Table 4), soft tissue injuries in 152 (55.9%) and osseous injuries in 41 (15.1%) horses. Iberian horses had a higher proportion of soft tissue injuries (66.7%) compared with Warmbloods (44.8%) (*Χ*^2^ = 7.54, *p* = 0.006). The prevalence of developmental injuries was similar for Warmblood and Iberian horses. There was an association between hypermetria and/or weakness and developmental disease (Fisher’s exact test *p* = 0.027). Horses with weakness had a higher proportion of developmental disease compared to horses with other or no gait abnormalities.

### 3.5. Follow-Up Outcome

In total, 34 horses (12.5%) were lost to follow-up. A total of 101 out of 238 horses (42.4%) maintained the same level of work as pre-injury or increased the level of work, 106 (44.5%) decreased the level of work, and 31 horses (13.0%) were retired because of orthopaedic injury. Overall, 101/238 horses (42.4%) had a good outcome, whereas 137/238 (57.6%) had a poor or bad outcome. A total of 14 of 112 horses (12.5%) with multilimbed lameness were retired, 52 (46.4%) decreased their level of performance and 46 (41.12%) had a good outcome (*p* = 0.86). Similarly, 14/104 horses (13.5%) with multifocal injuries were retired, 44 horses (42.3%) decreased performance level, and 46 horses (44.2%) maintained or increased pre-injury level of performance, *(p* = 0.83). There were no associations between age (*p* = 0.37), breed (*p* = 0.65), sex (*p* = 0.38), work level (*p* = 0.81), lameness grade (*p* = 0.51) or alignment of the distal phalanx (*p* = 0.21) and outcome. A total of 62 horses (22.8%) had routine daily access to paddock turnout. There was no significant association between access to paddock turnout (*p* = 0.91) and outcome.

There were no significant differences in outcomes (*p* = 0.64) between the Iberian and Warmblood breeds, with a good outcome in 44% (62/141) of Warmblood and 40% (36/90) of Iberian horses. In total, 45 of 90 Iberian horses decreased their performance (50.0%) compared with 58/141 Warmbloods (41.1%), and 21 of 141 Warmblood horses (14.9%) were retired because of orthopaedic disease compared with 9/90 Iberian horses (10%).

#### 3.5.1. Anatomical Site of Injury and Follow-Up Outcome

Of the twenty-three horses with combined injuries in the foot, one was lost to follow up. In total, 15/22 (68.2%) decreased their work level or were retired versus 7 (31.8%) that maintained or increased their level of performance. Of the 20 horses with palmar foot pain, 3 were lost to follow up; 3/17 horses were retired (17.7%), 6/17 (35.3%) had a poor outcome and 8/17 had a good outcome (47.1%). Of the 65 horses with dorsal foot pain, 11 were lost to follow up; 5/54 (9.3%) were retired, 24/54 (44.4%), had a poor outcome and 25/54 (46.3%) maintained or increased their level of performance.

Outcome data for each anatomical area are summarised in Table 5. Overall, 40/93 (43%) horses with foot pain had a good outcome. In total, 27 of 68 horses (29.0%) with fetlock region pain had a good outcome, compared with 34 of 78 horses (43.6%) with metacarpal or metatarsal pain and 19 of 51 (37.3%) horses with spinal pain.

There was an association between abnormalities in the caudal cervical region and outcome (*p* = 0.021). In total, 4 out of 9 (44.0%) horses with caudal cervical problems had a good outcome compared with only 29/81 horses (36.0%) with other problems.

#### 3.5.2. Type of Injury and Follow-Up Outcome

There were no significant differences between outcome and soft tissue injuries (*p* = 0.33), osteoarthritis (*p* = 0.91), bone injury (*p* = 0.42), or developmental disease (*p* = 0.30). Of the 31 horses with developmental disease, 45% had a good outcome, 32% decreased in level and 23% were retired due to orthopaedic injury.

#### 3.5.3. Neurological Dysfunction and Follow-Up Outcome

There was no significant difference in outcome for horses with hypermetria and weakness, hypermetria or weakness alone or for horses with no neurological gait abnormality (*p* = 0.65). In total, 15 of 39 horses (38.5%) with hypermetria alone had a good outcome, compared with 4/6 (38.5%) horses with hypermetria and weakness, 1/3 horses (33.35) with weakness alone and 15/39 (36.2%) horses with no neurological dysfunction.

## 4. Discussion

### 4.1. Results Related to Hypotheses

This retrospective study reports the prevalence of injuries and outcome for 272 dressage horses seen either as a first opinion or referred for investigation by a specialist. In accordance with the first hypothesis, neither multilimbed nor multifocal injuries influenced follow-up outcome despite being common. However, contrary to the second hypothesis Iberian horses had significantly more soft tissue injuries than Warmbloods, although there were no differences in overall follow-up outcome between breeds. Finally, contrary to the third hypothesis, access to paddock turnout before injury did not influence follow-up outcome.

#### 4.1.1. Multifocal and Multilimbed Lameness

Multifocal and multilimbed lameness is common in dressage horses [20] and in horses from other sports disciplines [29,30]. Several equine orthopaedic problems commonly occur bilaterally in sports horses, such as front foot pain [31], suspensory desmitis [30,32], fetlock injuries [33,34,35,36] and distal hock joint osteoarthritis [37]. In human athletes, it is recognized that the presence of an injury increases the likelihood of further injuries [38]. Compensatory overload of structures is expected in any species when trying to avoid loading of an injured area [38]. Moreover, accumulation of injuries through time is common in most athletes [38].

The effect of compensatory overload may depend on the severity of lameness. Mild injuries affecting several limbs if treated and managed appropriately may have no significant impact on outcome, whereas a severe unilateral lameness may be more likely to result in secondary injuries. Horses in the current study underwent in-depth investigations, often involving diagnostic anaesthesia of several limbs, to identify the sources of pain thus optimising subsequent management. Moreover, most horses showed only mild or moderate lameness, although the presence of lameness in more than one limb may result in the assignment of a lower lameness grade than that for a unilateral lameness [39]. This may explain why horses which presented for investigation of poor performance, that often had multilimbed lameness or spinal problems, had a lower median lameness grade than horses presented for lameness investigation.

The outcome results of the current study are in accordance with a previous report describing the long-term outcome of 70 dressage horses which were evaluated every six months at least five times. The presence of multilimbed lameness or multifocal injury was common and did not affect the follow-up outcome [20].

##### Management and Prevention of Multifocal and Multilimbed Injuries

Treatment of multifocal injury or multilimbed lameness can be challenging from a biomechanical perspective, especially when the structures involved have opposing functions such as the suspensory ligament and the DDFT. Pain in one structure may result in an increase in force in another structure. Although in horses with combined injuries one injury is often more severe than the other, giving biomechanical preference during treatment to one structure may result in deterioration of the less severe lesion. The aim is therefore to reach a compromise solution to avoid overload of either of the injured structures. The nature of the concurrent injuries may also influence the choice and timing of medication. For example, the use of corticosteroids for management of a suspensory ligament injury involving the enthesis may be contraindicated [40].

In the current study, partial improvement in lameness after diagnostic anaesthesia was usually not regarded as an endpoint of the investigation and further nerve blocks were performed until resolution of lameness in all limbs. This in-depth approach helps to ensure that all sources of pain are identified to optimise the chance of management success.

Failure to resolve lameness may result in the development of compensatory patterns of locomotion [41,42], resulting in overload of other limbs. It was previously observed that over time it was common for dressage horses to develop sequential patterns of lameness after a first lameness episode and thoracolumbosacroiliac region problems [20].

#### 4.1.2. Breed Differences

The higher prevalence of soft tissue injuries in Iberian horses compared with Warmblood horses may be multifactorial in origin, including genetic predisposition, conformation and gait characteristics. Genetic susceptibility to suspensory desmitis has previously been reported in other breeds [43,44,45] and merits further investigation in Iberian horses.

Biomechanical differences in gait between breeds have been reported [45]. Purebred Spanish Horses have an increased stride frequency compared with Warmbloods, but their tendency for increased carpal flexion may affect both propulsive and impact forces. Differences between extended trot and extended canter in Iberian horses versus Warmblood horses have not been investigated, but extended trot in Warmblood breeds resulted in greater load on the suspensory apparatus compared with collected trot [14]. An expressive extended trot is favoured in showing classes for PRE horses, and dressage horses in Spain have often previously competed in showing classes.

Conformation of the distal limb has also been correlated with soundness and performance [46,47,48,49] and it is well recognised that some limb deviations may predispose to certain injuries. Purebred Spanish horses have a considerable prevalence of angular limb defects, and their heritability has been reported to be substantial [17,50,51]. This might influence their susceptibility to soft tissue injuries [47]. Also, the high prevalence of angular deformities in the distal limb in PRE horses and the common hoof conformation with high heels (unpublished data) may affect risk of injury of the collateral ligaments of the interphalangeal joints, suspensory ligament branches and origin of the suspensory ligament.

Body condition score was not assessed in the current study; however, it is well-recognised that Iberian horses tend to be overweight [52]. Obesity has been recognised as a risk factor for injury in dressage horses [3] and specifically for suspensory ligament injury [32].

Although Iberian horses were at higher risk of soft tissue injury than Warmblood horses in the current study, no difference between breeds was observed in a previous study [20] in which there was a higher proportion of professionally produced horses working at a higher level. These horses may have a different propensity for injury.

#### 4.1.3. Paddock Turnout

Availability of paddock turnout did not influence the follow-up outcome, nonetheless, turn out in a paddock of suitable size may provide constant movement necessary for appropriate adaptation of healed tissue [3]. The posture for grazing requires spinal flexion and may promote epaxial muscle development; irregular terrain may enhance proprioceptive function. Moreover, mental relaxation and social interaction with other compatible horses are likely to improve both behaviour and mental state [53].

### 4.2. Horse Age

The median age of horses in the current study was 8 years, which is similar to many other studies investigating injuries in sports horses [31,32,33,34,35,37,54,55,56]. However, there were two peaks of injury occurrence at 4 to 5 and 8 and 9 years of age, respectively. Many dressage horses start their main training at 4 years of age, some in preparation for young horse classes. Overly rapid progression in training and repetitive training may result in insufficient time for the musculoskeletal system to adapt adequately without sustaining injury. In an unrelated study, young horses up to 5 years of age were at more risk of multiple suspensory injuries compared with horses 6 years of age or older [32]. In the current study, the prevalence of suspensory injuries was high which may in part account for the injury peak in young horses. In human athletes there is an increase in injury prevalence during training when new exercises have been introduced [54]. Dressage horses that are training towards higher levels are asked to work with increased collection, and more demanding lateral movements are introduced, flying changes, and ultimately piaffe, passage and canter pirouettes. This commonly starts at 8 to 9 years of age, and some horses may not have sufficient musculoskeletal strength and coordination to cope with the forces generated by these movements. Moreover, some riders and trainers fail to understand that excessive training may be detrimental both physically and mentally, do not appreciate that recovery time is required between intense training sessions, and may misunderstand behaviors that reflect discomfort rather than misinterpretation of the rider’s cues.

### 4.3. Distribution of Sources of Pain and Prevalence

#### 4.3.1. Foot Pain

As previously reported in dressage horses [3], in the current study foot pain was the most prevalent area for injury. Osteoarthritis of the DIP joint was the most common diagnosis with a prevalence of 76.8%. This high frequency is difficult to compare with previous studies of foot-related pain which have usually focused on referral populations of horses [31,55,56,57,58] rather than horses in first opinion practice.

#### 4.3.2. Suspensory Ligament Injury

In the current study, metacarpal/metatarsal region pain was the second most common diagnosis and mostly due to suspensory desmitis, with proximal suspensory desmitis occurring in 21% of all horses.

Among the risk factors reported, early training of extended trot and repetitions in young horses and excessive work in circles are the most common scenarios for suspensory ligament injuries. As previously reported, there is a high recurrence of suspensory desmitis in dressage horses [20]. Mild and bilateral injuries may not be recognised by owners unless a horse is lunged or routinely monitored for lameness. Muscle fatigue, especially in young animals may result in missteps which are common during training. Lungeing may be performed before ridden exercise to avoid undesirable behaviors and can contribute to fatigue. Exuberant horses may also lack balance when lunged, potentially increasing injury risk. Poor arena surface maintenance has been reported as a risk factor for lameness in dressage horses [3,4]. Irregular or too deep surfaces may favor hyperextension of the fetlock joint. Excessive compression forces and stress shielding have been reported to be one of the causes in human enthesitis, in addition to tensile loads [59].

There was a positive association between metacarpal or metatarsal region pain (often suspensory ligament injury) and fetlock region pain. The relationship between fetlock pathology and suspensory desmitis has previously been recognized [34]. During extension of the metacarpophalangeal or metatarsophalangeal joints the suspensory ligament is subjected to high tensile loads [60]. The suspensory branches effectively become part of the articular surface and provide palmar or plantar support for the joint. Fetlock extension is repeated during many exercises in dressage and suspensory branch injury may reflect repetitive overload. The distal two-thirds of the suspensory branches are subsynovial to the palmar (plantar) recesses of the fetlock joint capsules [61]. Suspensory branch injuries with exposure of fibers in contact with synovial environments might precipitate an inflammatory cascade within the joint, predisposing to osteoarthritis. Alternatively, osteoarthritis may precede signs of suspensory branch desmitis. Altered loading of the fetlock joint to reduce pain may result in increased strain on the suspensory apparatus predisposing to injury. It is therefore suggested that whenever dealing with fetlock region pain in dressage horses the fetlock joint and the suspensory apparatus should be carefully evaluated using diagnostic imaging and appropriate treatment instigated. In the current study, fetlock region pain was the third most common source of pain and is one of the most common injuries reported in Warmblood horses [62].

The incidence of hindlimb suspensory desmopathy in the current study was low compared with other reports [2,32,63,64,65]. This may reflect the low prevalence of hindlimb lameness in this study (28%) because of the failure of trainers/owners/riders to recognise hindlimb lameness, as previously reported [66].

#### 4.3.3. Spinal Pain

Spinal pain occurred in 22% of horses overall, and prevalence increased with age, similar to previous reports [3,20]. Entheseous new bone on the caudal aspect of the occiput was not included as a diagnostic criterion because this occurs in many dressage horses without associated clinical signs [67].

It is well recognised that enlargement of the articular processes and other radiological findings in the caudal cervical region can be present in horses with no associated clinical signs [68,69,70,71]. Nonetheless, in the current study caudal cervical pain was identified in 5.9% of horses, resulting in either pain-induced cervical dysfunction or forelimb lameness. In a recent case-control study, although dressage horses were the largest group, dressage horses with neck pathology (neck pain or stiffness, forelimb lameness or neurological dysfunction) were under-represented and show jumping horses were over-represented [71]. Dressage horses must flex laterally and in the sagittal plane and rotate the caudal cervical vertebrae, which may be restricted by enlarged articular processes. However, the intervertebral foramina are smallest with the caudal cervical region in extension [72], as occurs when show jumpers are landing, which may result in nerve root compression.

In the current study, there was a moderate prevalence of thoracolumbar (10%) or lumbosacroiliac (10%) region pain or both (3%). During dressage exercises, rapid flexion and extension of the thoracolumbosacral region are required [10] as well as lateral bending and axial rotation [73]. Collection demands increased thoracic and lumbosacral flexion, and in canter there is an active concentric contraction of the trunk muscles [10], with the iliopsoas muscles having a major role. After the hindlimb impulsion phase, the lumbosacral region extends pushing the horse forward followed by flexion during the suspension phase. In the current study, clinical signs associated with thoracolumbosacroiliac region pain were worst when ridden as previously reported [74,75,76,77], highlighting the importance of evaluating dressage horses being ridden and performing their full repertoire of movements [20,74,76,78].

Adequate muscle function is clearly important for optimal performance; spinal pain is not only localized to the osseous tissues; paraspinal muscle contractures and muscle pain commonly occur concurrently [77,79]. As a result of reduced movement in affected areas, muscle tissue loses both elasticity and size of muscle fibers. Atrophy of the multifidus muscles occurs adjacent to osseous pathology in both horses [77,80], and humans [79]. Muscle atrophy of the paraspinal muscles is a common clinical sign [29,71,76,81] and treatment must focus not only on pain relief but also on restoration of neuromuscular pathways to promote normal muscle function [74,82,83].

Due to the high prevalence of lumbosacroiliac joint region pain in dressage horses, the routine use of rectal ultrasonography routinely is recommended [75,84,85,86,87] to determine the optimal treatment and management for each horse based on the findings. With primary involvement of the lumbosacral joint, the treatment aims to restore function by decreasing pain but avoiding any aggressive mobilisations or forcing lumbosacral flexion [88]. However, with primary sacroiliac joint region pathology without involvement of the lumbosacral joint, after local treatment lumbosacral flexion and extension are recommended [88]. Follow up ultrasonographic examination over time is recommended because the prevalence of lumbosacroiliac joint region problems increases with age [20,84,85,89].

#### 4.3.4. Neurological Dysfunction

In contrast to a previous study [20] in which horses with hypermetric and weak gaits were more likely to have a poor outcome compared with both horses with no neurological gait abnormality or those with a hypermetric gait, hypermetria and weakness did not influence follow-up outcome in the current study. This may reflect the small proportion of horses in the study with neurological dysfunction.

### 4.4. Prevention of Injury

There is a lack of knowledge regarding prevention of orthopaedic injuries in both human and equine athletes. Even in human sports medicine, techniques that reduce injury prevalence lack scientific support [38]. Changes in equipment, training techniques, nutrition and psychological measures had poor evidence of efficacy among human athletes [38]. However, alterations in exercise patterns may be an effective method to prevent injuries [38]. Promotion of muscle strength and support has diminished the incidence of certain lesions in human athletes [38]. Equine athletes require time for tissue adaptation to newly introduced forces, and time for modelling and repair after intensive efforts [90]. Slow transitions in loads are generally recommended [90].

Injuries in pre-elite human and equine athletes are common, and these injuries can limit potential [91]. Previous injuries predispose athletes to further injury [38]. Therefore, routine examination of dressage horses and early recognition of injuries have the potential to improve the outcome [20].

### 4.5. Limitations of the Study

This clinical study had some limitations with some inherent biases based on the population of horses presented for investigation (65% amateur or young horses), economic constraints of some owners which may have influenced treatment and outcome, and the diagnostic, treatment and management strategies of the investigating veterinarian. The methodologies may have evolved over time. The duration of lameness prior to investigation was not known, but this information is reliant on rider recognition of lameness or a decline in performance and is unreliable. In some situations, amateur and U25 horses may be schoolmasters which had previously trained or competed at a higher level and therefore have the associated cumulative work history. However, this was not the case in the current study.

Body condition score and body weight were not recorded. Potential biases of the owner, trainer and rider may have affected their perceptions about performance and their decisions about subsequent physical activity, participation in competitions, and retirement. The duration of follow-up was variable, and a survival analysis was not performed. While recommendations about horse management, training strategies, farriery and available footing, cross training, tack fit and nutrition were discussed, owner compliance could not be controlled, which may have influenced outcomes.

## 5. Conclusions

In this population of dressage horses, multifocal pain and multilimbed lameness were prevalent, but neither were associated with follow-up outcome. Foot, fetlock, metacarpal and metatarsal and spinal pain were common. There was an association between fetlock and metacarpal/metatarsal pain; thus, when pain causing lameness is localised to one of these areas, imaging of both regions is recommended. Ridden exercise is an important part of the clinical assessment and should include collection and canter, especially for the identification of spinal region pain. Diagnostic anaesthesia, together with the results of an in-depth clinical assessment, are important for recognition of all problems and for the development of the most suitable treatment and management strategies.

## Figures and Tables

**Table 1:** Summary of different treatments administered to 272 dressage horses after definitive diagnosis of the cause(s) of lameness or poor performance. * Triamcinolone (6–12 mg/joint variable according to severity and concomitant treatments), betamethasone 6–18 mg/joint, methylprednisolone acetate (only used in spine—20 mg/site), dexamethasone 6–18 mg/joint (variable products for each corticosteroid). If several joints were treated, doses were adjusted to maximum safe dose.

Osteoarthritis	Intraarticular treatments: Corticosteroid therapy (CS) variable according to injury, horse variables (for example, withdrawal time, body condition, age) and availability * CS+ sodium hyaluronan Polyacrylamide gel in cases of considerable subchondral bone involvement or extensive periarticular modelling 48 h rest+ decreased painful exercises for 15 days
Subchondral Bone Injury	Bisphosphonates (e.g., tiludronate) Radial Pressure Wave Therapy (RPWT) weekly or every 15 days for a total of 3–4 treatments Period of rest 15–30 days with hand walking + paddock Gradual return to training variable according to injury and response to treatment
Large (≥50% of the total cross-sectional [CSA] area) core lesion of a tendon or ligament (non-synovial environment)	Intralesional umbilical cord cultured mesenchymal stem cell therapy Stem cell treatment + RPWT weekly or every 15 days for a total of 3–4 treatments according to ultrasonographic progression Hand walking until ultrasonographic improvement (minimum of 3 months commonly for severe injuries) followed by gradual return to exercise monitored by ultrasonographic control
Small (>25% < 50% of CSA) core or diffuse lesion of a tendon or ligament	RPWT weekly or every 15 days for a total of 3–4 treatments according to ultrasonographic progression Hand walking until ultrasonographic improvement If mild—continue training but with restriction of exercise
Tenosynovitis	Surgical debridement of lesions and tenoscopic lavage if moderate to severe (marked distension, synovitis and lesion accessible by tenoscopy) RPWT if mild lesions or lesions not accessible or in contact with synovial fluid Hand walking until ultrasonographic improvement

**Table 2:** Primary diagnosis for the anatomical site of injury within the foot region (n = 108) during investigation of lameness in 272 dressage horses. The total number of diagnoses exceeds 108 because some horses had more than one injury. Distal phalanx includes horses with primary bone trauma (abnormal signal intensity on magnetic resonance images) and those with laminitis (the dorsal aspects of the hoof capsule and distal phalanx were not aligned). DIP = distal interphalangeal.

Anatomical Area/Injury	Number	Percentage
Distal interphalangeal (DIP) joint osteoarthritis	83	76.8
Collateral desmitis of DIP joint	18	16.7
Ungular cartilages	10	9.3
Podotrochlear apparatus	10	9.3
Deep digital flexor tendon	13	12.0
Distal phalanx	26	24.1

**Table 3:** Distribution of soft tissue injuries in the metacarpal and metatarsal and fetlock regions in 272 dressage horses undergoing investigation of lameness or poor performance. The percentage refers to the proportion of 272 horses.

Anatomical Structure	Number	Percentage
Forelimb suspensory (SL) ligament branch(es)	16	5.9
Forelimb proximal suspensory desmitis	56	20.6
Forelimb superficial digital flexor tendon	15	5.2
Forelimb accessory ligament of the deep digital flexor tendon	5	1.8
Hindlimb SL branch(es)	16	5.9
Hindlimb proximal suspensory desmopathy	4	1.5

**Table 4:** Distribution of joints with radiological or ultrasonographic evidence of osteoarthritis which was causing pain and/or lameness in 272 dressage horses in training or competing. DIP = Distal interphalangeal joint. PIP = Proximal interphalangeal joint. MCP = Metacarpophalangeal joint. MTP = Metatarsophalangeal. Distal tarsal joints refer to centrodistal and tarsometatarsal joints. Carpal joints refer to antebrachiocarpal, middle carpal and carpometacarpal joints.

Joint	Number of Horses	Percentage
DIP and PIP joints	83	30.5
MCP/MTP joints	71	26.1
Distal tarsal joints	26	9.6
Tarsocrural joint	8	2.9
Carpal joints	8	2.9
Femorotibial and femoropatellar joints	21	7.7
Caudal cervical articular process joints	19	7.0
Lumbosacroiliac joints	36	13.2

**Table 5:** A summary of available follow-up data for 238 dressage horses according to injury site. Maintained performance = work level at time of the initial examination maintained or increased. Decreased performance = work level decreased compared with pre-injury. Retired = retired because of injury. Cranial cervical = occiput to fourth cervical vertebra. Caudal cervical = fifth cervical vertebra to first thoracic vertebra.

Anatomical Area	Injury	Maintained Performance	Decreased Performance	Retired	Chi-squared/Fisher’s Exact Test *p*-Value *
Foot	Yes (n = 93)	40 (43.0%)	39 (41.9%)	14 (15.1%)	0.696
	No (n = 145)	61 (42.1%)	67 (46.2%)	17 (11.7%)	
Fetlock	Yes (n = 68)	27 (9.0%)	29 (42.6%)	12 (17.7%)	0.406
	No (n = 170)	74 (43.5%)	77 (45.3%)	19 (11.2%)	
Metacarpal/metatarsal	Yes (n = 78)	34 (43.6%)	36 (46.2%)	8 (10.3%)	0.675
	No (n = 160)	67 (41.9%)	70 (43.8%)	23 (14.4%)	
Cranial cervical	Yes (n = 1)	0	0	1 (100.0%)	-
	No (n = 40)	12 (30.0%)	25 (62.5%)	3 (7.5%)	
Caudal cervical	Yes (n = 9)	4 (44.4%)	2 (22.2%)	3 (33.3%)	0.021
	No (n = 81)	29 (35.8%)	47 (58.0)	5 (6.2%)	
Thoracolumbar	Yes (n = 23)	8 (38.1%)	12 (57.1%)	1 (4.8%)	0.872
	No (n = 69)	25 (36.2%)	37 (53.6%)	7 (10.1%)	
Lumbosacroiliac	Yes (n = 23)	7 (25.9%)	13 (61.9%)	1 (4.8%)	0.872
	No (n = 67)	24 (35.8%)	36 (53.7%)	7 (10.4%)	

* depicts relationship between anatomical area and follow-up outcome.

## Data Availability

Anonymized data are available from the authors upon reasonable request.

## Supplementary Materials

The following supporting information can be downloaded at: https://www.mdpi.com/article/10.3390/ani15202972/s1, Table S1 An overview of the standard radiographic projections acquired for each anatomical region.
