# Supplementary material for: Orthopaedic Injuries in 272 Dressage Horses: A Retrospective Study
Source: Animals (Basel). 2025 Oct 14;15(20):2972. doi: 10.3390/ani15202972 (PMC12560884; doi:10.3390/ani15202972)
Supplement: Supplementary file 1 [file animals-15-02972-s001.zip › animals-3841715-supplementary.docx]

Supplementary Table S1 An overview of the standard radiographic projections acquired for each anatomical region [1].

LM = lateromedial; DPr-PaDiO = dorsoproximal-palmarodistal oblique; DPa = dorsopalmar; PaPr-Pa DiO = palmaroproximal-palmarodistal oblique; D45°M-PaLO = dorsal 45° lateral-palmaromedial oblique; D45°M-PaLO = dorsal 45°medial-palmarolateral oblique; D10-15°Pr-PaDiO = dorsal 10-15° proximal – palmarodistal oblique; DPl = dorsoplantar; D45°L-PlMO = dorsal 45°lateral-plantaromedial oblique; D45°M-PlLO = dorsal 45°medial-plantaromedial oblique; Cd 15°Pr-Cr DiO = caudal 15°proximal – craniodistal oblique

| Anatomical area | Images acquired | Comments |
| --- | --- | --- |
| Foot | LM, DPa, DPr-PaDiO, PaPr-PaDiO | After shoe removal, cleaning the foot and packing the frog cleft & sulci |
| Metacarpophalangeal joint | LM or LM (Flexed), D10-15°Pr-PaDiO, D45°L-PaMO, D45°M-PaLO |  |
| Carpus | LM (Flexed), DPa, D45°L-PaMO, D45°M-PaLO |  |
| Metatarsophalangeal joint | LM, DPl, D15-20°Pr-PlDiO, D45°L-PlMO, D45°M-PlLO | With the metatarsal region vertical |
| Tarsus | L5°Pr-MDiO, DPl, D45°L-PlMO, D45°M-PlLO | For evaluation of the plantar cortex of the third metatarsal bone L5°Pr5-10°Pl-MDiDO |
| Stifle | LM (flexed), Cd 15°Pr-CrDiO |  |

1. Butler, J.A.; Colles, C.M.; Dyson, S.J.; Kold, S.E.; Poulos, P.W. *Clinical Radiology of the Horse*; 4th ed.; Wiley-Blackwell: Oxford, UK, 2016
